# The genotypic and phenotypic spectrum of PIGA deficiency

**DOI:** 10.1186/s13023-015-0243-8

**Published:** 2015-02-27

**Authors:** Maja Tarailo-Graovac, Graham Sinclair, Sylvia Stockler-Ipsiroglu, Margot Van Allen, Jacob Rozmus, Casper Shyr, Roberta Biancheri, Tracey Oh, Bryan Sayson, Mirafe Lafek, Colin J Ross, Wendy P Robinson, Wyeth W Wasserman, Andrea Rossi, Clara DM van Karnebeek

**Affiliations:** Centre for Molecular Medicine and Therapeutics, Vancouver, Canada; Department of Medical Genetics, University of British Columbia, Vancouver, Canada; Treatable Intellectual Disability Endeavour in British Columbia, Vancouver, Canada; Division of Biochemical Diseases, Department of Pediatrics, BC Children’s Hospital, University of British Columbia, Vancouver, Canada; Division of Hematology, Oncology & BMT, Department of Pediatrics, BC Children’s Hospital, University of British Columbia, Vancouver, Canada; Biochemical Genetics Laboratory, Department of Pathology, BC Children’s Hospital, University of British Columbia, Vancouver, Canada; Child & Family Research Institute, Vancouver, BC Canada; Department of Paediatric Neurology, Children’s Hospital Oxford, John Radcliffe Hospital, Oxford, UK; Department of Neuroradiology, Istituto Giannina Gaslini, Via Gerolamo Gaslini 5, I-16147 Genoa, Italy

**Keywords:** Intellectual disability, Epileptic encephalopathy, Hypotonia, Dysmorphism, Multi-organ involvement, Genomics, Intramyelin edema, Glycosylphosphatidylinositol, Lipoprotein lipase, Alkaline phosphatase, Iron

## Abstract

**Background:**

Phosphatidylinositol glycan biosynthesis class A protein (PIGA) is one of the enzymes involved in the biosynthesis of glycosylphosphatidylinositol (GPI) anchor proteins, which function as enzymes, adhesion molecules, complement regulators and co-receptors in signal transduction pathways. Until recently, only somatic *PIGA* mutations had been reported in patients with paroxysmal nocturnal hemoglobinuria (PNH), while germline mutations had not been observed, and were suspected to result in lethality. However, in just two years, whole exome sequencing (WES) analyses have identified germline *PIGA* mutations in male patients with XLIDD (X-linked intellectual developmental disorder) with a wide spectrum of clinical presentations.

**Methods and results:**

Here, we report on a new missense *PIGA* germline mutation [g.15342986C>T (p.S330N)] identified via WES followed by Sanger sequencing, in a Chinese male infant presenting with developmental arrest, infantile spasms, a pattern of lesion distribution on brain MRI resembling that typical of maple syrup urine disease, contractures, dysmorphism, elevated alkaline phosphatase, mixed hearing loss (a combination of conductive and sensorineural), liver dysfunction, mitochondrial complex I and V deficiency, and therapy-responsive dyslipidemia with confirmed lipoprotein lipase deficiency. X-inactivation studies showed skewing in the clinically unaffected carrier mother, and CD109 surface expression in patient fibroblasts was 57% of that measured in controls; together these data support pathogenicity of this mutation. Furthermore, we review all reported germline *PIGA* mutations (1 nonsense, 1 frameshift, 1 in-frame deletion, five missense) in 8 unrelated families.

**Conclusions:**

Our case further delineates the heterogeneous phenotype of this condition for which we propose the term ‘PIGA deficiency’. While the phenotypic spectrum is wide, it could be classified into two types (severe and less severe) with shared hallmarks of infantile spasms with hypsarrhythmia on EEG and profound XLIDD. In severe PIGA deficiency, as described in our patient, patients also present with dysmorphic facial features, multiple CNS abnormalities, such as thin corpus callosum and delayed myelination, as well as hypotonia and elevated alkaline phosphatase along with liver, renal, and cardiac involvement; its course is often fatal. The less severe form of PIGA deficiency does not involve facial dysmorphism and multiple CNS abnormalities; instead, patients present with milder IDD, treatable seizures and generally a longer lifespan.

## Background

Glycosylphosphatidylinositol (GPI) is a glycolipid that is synthesized and transferred to proteins in the membrane of the endoplasmic reticulum [1]. Biogenesis of GPI anchored proteins is a conserved post-translational mechanism in eukaryotes and is important for attaching these proteins to the cell membrane, for protein sorting, trafficking, and dynamics [1], and plays an essential role in embryogenesis, neurogenesis, immune responses, and fertilization [2-6]. To date, more than 20 phosphatidylinositol glycan biosynthesis protein (PIG) subclasses have been found to be involved in GPI anchor biosynthesis and remodeling, and more than 150 proteins carry GPI anchors [1]. An increasing number of human diseases have been discovered to be due to mutations in GPI biosynthesis genes.

*PIGA* (MIM 311770) encodes one of the seven proteins involved in the transfer of N-acetylglucosamine (GlcNAc) from UDP-N-acetylglucosamide (UDP-GlcNAc) to phosphatidylinositol (PI) to form GlcNac-PI [1]. This is the first step of GPI anchor biosynthesis and takes place on cytoplasmic side of the endoplasmic reticulum [1]. The human *PIGA* gene is located on chromosome Xp22.2. It spans 162 kb and the longest transcript (NP_002632.1) encodes for a protein of 484 amino acids expressed in a wide variety of tissues including brain, liver, heart, and blood cells [7]. Somatic *PIGA* mutations had been well documented in PNH [MIM 300818], an acquired hemolytic disease that manifests after clonal expansion of hematopoietic cells with somatic *PIGA* mutations, where loss of CD55 and CD59 on erythrocytes causes complement-mediated lysis [8-12]. Unlike somatic *PIGA* mutations, germline mutations had not been observed until recently, and based on experiments in mice [2] and in both murine [13] and human embryonic stem cells [14] it had been proposed that germline *PIGA* mutations were lethal. In 2012, using an X-chromosome exome next-generation sequencing screen, Biesecker and colleagues identified a *PIGA* germline nonsense mutation in two siblings with an early epileptic encephalopathy with hypotonia, brain anomalies (myelination abnormalities and a thin corpus callosum), cleft palate, cardiac anomalies and early death [15]. Recently, four additional clinical reports were published on patients with germline *PIGA* mutations depicting a wide spectrum of phenotypes and clinical diagnoses [7,16-18], including West syndrome [18], Multiple congenital anomalies-hypotonia-seizures syndrome 2 (MCAHS2) [17,18] and Ferro-Cerebro-Cutaneous syndrome (FCCS) [16]. Here, we report a male patient with MRI brain abnormalities that resemble those of infants with maple syrup urine disease (MSUD), multi-organ involvement, therapy-responsive dyslipidemia, and reductions of mitochondrial respiratory complexes I and V on Blue Native Gel (BNG) analysis. Using WES, we identified in this patient a new missense *PIGA* germline mutation (g.15342986C>T, c.989G>A [NM_002641], [p.S330N]), which will be referred to as c.989G>A (p.S330N). We also review a total of 8 mutations from 9 unrelated families, summarize clinical findings, discuss genotype–phenotype correlations and identify common features that may be used to guide clinical identification of patients with germline PIGA mutations.

## Methods

This study was approved as part of our TIDEX gene discovery project by the Ethics Board of the Faculty of Medicine of the University of British Columbia (UBC IRB approval H12-00067). Parents provided written informed consent for publication of this report.

### Case report

**Figure 1:**
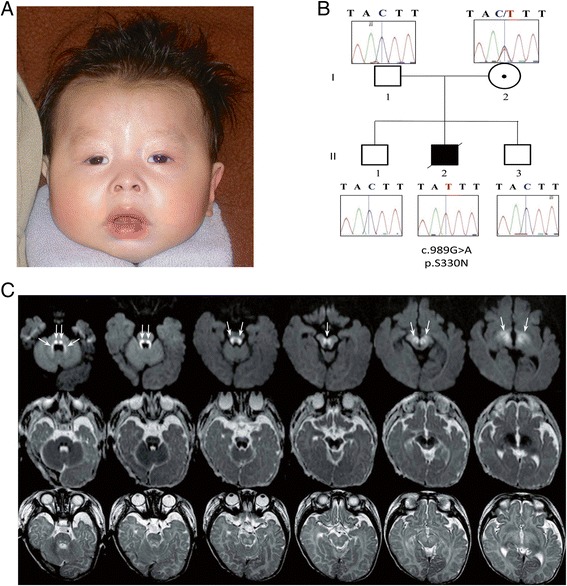
**Patient images and pedigree of the family. (A)** Facial features of the index patient at age 14 months. **(B)** Pedigree of the family with new c.989G > A germline *PIGA* mutation. Sanger sequence verification is shown next to each member of the family. The deceased index was confirmed to be hemizygous for the variant, while mother was confirmed to be a carrier; father and two unaffected brothers were confirmed to have a normal copy of the gene. **(C)** MRI at age 4 months. Upper row, axial diffusion-weighted images (DWI), b = 1000 s/mm^2^; middle row, corresponding apparent diffusion coefficient (ADC) maps; bottom row, axial T2-weighted images. There is restricted diffusion, evidenced by high signal on DWI (arrows) and corresponding low signal on the ADC maps, at the level of the ponto-medullary tegmentum, superior cerebellar peduncles, ventral midbrain, subthalamus, and inferior striatum. The same regions show abnormally elevated signal on the T2-weighted images. The abnormalities selectively involve regions that are normally myelinated at this age and are consistent with intramyelin edema.

The male index (II:2) was the second child (Figure 1) of non-consanguineous healthy Chinese parents with unremarkable family history. Prenatal ultrasound showed macrosomia and increased nuchal thickness. At 31 weeks 6 days gestation the biparietal diameter (BPD) was 89 mm (97th centile), head circumference (HC) was 322 mm (91st centile), abdominal circumference (AC) 330 mm (>99th centile), and femur length (FL) was 64 mm (86th centil) using B.C. Women’s Health Centre standard measurements. At 22 weeks 3 days gestation BPD was 59 mm (89th centile), HC was 215 mm (81st centile), AC was 213 mm (>99th centile), and FL 42 mm (89th centile). Nuchal fold measurement at 20 weeks 3 days was 6.9 mm. Maternal serum alpha-fetal protein (AFP) at 16 weeks 0 days was 49.1 ug/L which is 1.37 multiples of the mean (MoM). Amniotic fluid AFP was 22.0 mg/L which is 3.61 MoM (with a risk assessment for open spina bifida of 1:11). Acetylcholinesterase electrophoresis appeared to be normal. Prenatal amniocentesis revealed normal male karyotype (46,XY). The boy was born at 38 weeks by Caesarean section due to breech position with Apgar scores 5 and 9. His birth weight was 3975 g (>99th centile), length was 51 cm (~75th centile) and head circumference 36.0 cm (98th centile). Dysmorphism was noted at birth but became more apparent at a later age. Phototherapy was administered during one week for unconjugated hyperbilirubinemia.

The onset of epilepsy was 2.4 months; he had intractable seizures, which were initially classified as infantile spasms (EEG showed modified hypsarrhythmia), evolving one month later to myoclonic seizures (EEG showed suppression-burst-like pattern). He showed acquired microcephaly at 8.7 months with an occipital frontal circumference (OFC) of 43 cm (~3rd centile). On examination at 14 months (Figure 1A), our patient showed plagio-brachycephaly with the flattening of the right occiput greater than the left and a prominent right side of the face, giving the appearance of facial asymmetry. There was a frontal hair upsweep with unruly scalp hair and poorly defined eyebrows laterally. He had a round face with flattening of the lateral profile, a broad forehead with shallow orbital ridges and supraorbital indentations, most likely due to poor muscle function in the temples. There was glabellar fullness, bilateral ptosis with hypertelorism (intercanthal distance 3.0 cm; +2SD), upslanted short palpebral fissures (palpebral fissure length 2.0 cm; −2.0 SD), and an interpupillary distance of 4.95 cm (90th centile). The outercanthal distance was 7.0 cm (−1 SD), within normal limits the short palpebral fissures correct for the hypertelorism. The nose was short (2.6 cm; −2 SD) with a low bridge, prominent lateral nasal cartilage and anteverted nares. Ears were of normal size, placement and form. Philtrum appeared relatively long (measured 1.2 cm) and smooth. There was retrognathia, a tented upper lip with thickened alveolar overgrowth giving the appearance of a high arched palate (intact). He showed decreased facial expression. Redundant skin at the neck was noted, along with contractures of the small and large joints of upper and lower extremities; no pigmentation or other cutaneous abnormalities were noted. From a neurologic perspective there was profound global developmental delay with hyperekplexia, axial hypotonia, peripheral hypertonia, rigidity and abnormal cry. There was decreased range of motion of all major joints including the shoulders, elbows, wrist and fingers, as well as hips, knees and ankles. He had hand splints for camptodactyly, deep palmar creases (because of his fisting) and minimal movement of his fingers.

Brain MRI at presentation (age 4 months) revealed signal abnormalities with restricted diffusion at level of the brainstem tegmentum, superior cerebellar peduncles, subthalamus, and ventral striatum (Figure 1C), while follow-up studies at 10 months and 2.5 years showed progressive, severe cerebral and cerebellar atrophy associated with diffuse leukoencephalopathy and thinning of the corpus callosum. Serial MR spectroscopy showed initially mildly elevated lactate peaks, normal on subsequent imaging, as well as mild reduction of N-acetyl aspartate in the mid brain.

His heart was mildly enlarged and ECG and Holter revealed right ventricular hypertrophy and arrhythmia (AV block, Wenckebach type I). He also had moderate hepatomegaly, apnea, unilateral hydronephrosis with renal calculi, GI dysmotility which led to aspirations requiring G-tube feeding, visual motor impairment and retinal dystrophy (pale optic disc, flat macula, myopia, atrophic retina; no cherry red spot), moderate conductive and sensorineural hearing loss, stomatocytosis and hyperechoic liver. The child died at the age of 3.4 years of cardiac arrest; autopsy was not performed.

TORCH (toxoplasmosis, rubella, cytomegalovirus, herpes simplex, and HIV) screening was negative. A comprehensive metabolic investigation was initiated but largely uninformative, with the following exceptions: Plasma lipid profiling revealed markedly elevated triglycerides at 85.31 mM (reference 0.4-1.5) and cholesterol at 23.4 mM (reference 2.6-5.2), and absent post-heparin lipoprotein lipase activity suggesting a lipoprotein lipase deficiency. The abnormal lipid profile normalized quickly with the implementation of a medium-chain triglyceride enriched diet. Persistently elevated alkaline phosphatase levels were also noted, ranging from 364–649 U/L (Ref 110–320); calcium, phosphate and vitamin D were within normal limits.

BNG Analysis of a muscle biopsy at 10 months showed decreased amount of complex of I and V (at 30% and 10% of a tissue-matched control sample, respectively). These findings led to the initial clinical suspicion of a mitochondrial deficiency syndrome (lactates varied between normal – 3.8 umol/L) or lipid storage disorder, and investigations were pursued accordingly.

Further molecular investigations yielded normal results including array-CGH analysis for copy number variations (CNVs), mtDNA genome sequencing, targeted gene sequencing of *LPL, MECP2, ARX* and a number of nuclear encoded mitochondrial proteins. Elaborate biochemical testing was completed with essentially unremarkable results (ammonia, acylcarnitine profile, plasma amino acids, very long chain fatty acids, transferrin iso-electric focussing; urine organic acids, purines & pyrimidines, mucopolysaccharides, oligosaccharides, bile acids). The following enzymatic analyses yielded normal results: acid phosphatase, sphingomyelinase, arylsulphatase A, hexosaminidase A&B, biotinidase, chitotriosidase, galactosyl-ceramidase, beta-glucosidase, beta-galactosidase, cathepsin D, palmitoyl protein thio-esterase I, tripeptidyl peptidase I. Filippin staining studies in fibroblasts showed mildly atypical peri-nuclear vesicular accumulations of un-esterified cholesterol but were considered non-diagnostic. Sphingomyelinase enzymology and *NPC1* and *NPC2* sequencing were all unremarkable. Iron staining of muscle was negative.

### Whole exome sequencing

With a profound IDD and an abnormal biochemical phenotype, this patient met the inclusion requirements for our TIDEX (Treatable Intellectual Disability Endeavour exome sequencing) gene discovery study. We isolated genomic DNA samples from the peripheral blood of the patient as well as parents and two unaffected male siblings using standard techniques. WES was performed for the index patient and his unaffected parents using the Agilent SureSelect kit and Illumina HiSeq 2000 (Perkin-Elmer, Santa Clara, California, USA). An in-house designed bioinformatics pipeline [19] was used to align the reads to the human reference genome version hg19 and to identify and assess rare variants for their potential to disrupt protein function. The candidate variants were further confirmed using Sanger re-sequencing in all the family members. Deleteriousness of the candidate variants was assessed using Combined Annotation–Dependent Depletion (CADD) scores [20], PolyPhen-2 (http://genetics.bwh.harvard.edu/pph2/) [21] and SIFT (Sorting Intolerant From Tolerant; (http://sift.jcvi.org/) [22]. Protein alignment was generated using T-Coffee (http://www.tcoffee.org/) [23] and analyzed using GeneDoc http://www.nrbsc.org/gfx/genedoc/gdpaf.htm/). Only those variants predicted to be “functional” (missense, nonsense and frameshift changes, as well as in-frame deletions and splice-site effects) were subsequently screened under a series of inheritance models.

### X-inactivation studies

X-chromosome inactivation (XCI) was assayed in the unaffected mother using the allelic ratio of methylated alleles at the Androgen Receptor (*AR*) locus [24] as described previously [25]. The degree of allelic bias in terms of which X chromosome is inactivated can range from 50% (completely random) to 100% (completely skewed).

### Functional analysis

To provide evidence for pathogenicity of the identified mutation, patient and control skin fibroblasts were analyzed by flow cytometry for surface expression of CD109, a GPI-anchored protein. Cells were washed with PBS (without Ca2+ or Mg2+) (Gibco), detached using 5 mM EDTA (Gibco) in PBS and washed in 2% FBS (Gibco)/PBS. Cells were then stained for 20 minutes at 4°C with CD109-PE (BioLegend) and PE mouse IgG1, κ isotype control (BioLegend). Data were acquired using a BD™ LSR II flow cytometer and analyzed using FlowJo v8.8.4 (Tree Star). Protein expression was determined by Western blot analysis. Briefly, skin fibroblasts were harvested and lysed (cell lysis buffer, Cell Signaling) in the presence of protease inhibitor cocktails (Roche). Equal amounts of protein were separated by SDS-PAGE, transferred to PVDF membrane and blocked with 5% BSA. Protein expression was detected using an anti-PIG-A (clone H-6, Santa Cruz) primary antibody. After washing, bound antibody was detected with HRP-conjugated anti-mouse secondary antibody and Novex ECL chemiluminescent substrate (Invitrogen).

## Results

### New *PIGA* germline mutation in our patient with multisystem disease

**Figure 2:**
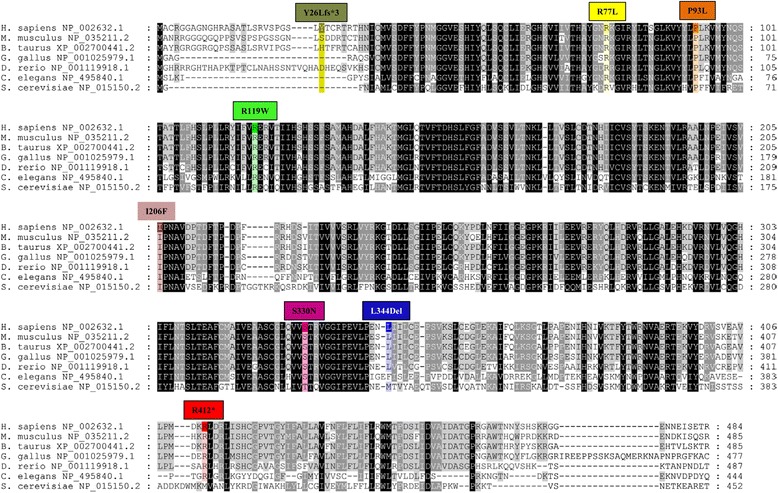
**A multi-sequence alignment of PIGA proteins and distribution of all known germline mutations.** The PIGA protein sequences were generated using depicted transcript identifiers from: *Homo sapiens* (human), *Mus musculus* (mouse), *Bos taurus* (cow), *Gallus gallus* (chicken), *Danio rerio* (zebrafish), *Caenorhabditis elegans* (worm) and *Saccharomyces cerevisiae* (yeast). Protein alignment was generated using T-Coffee [23] and analyzed using GeneDoc.

**Figure 3:**
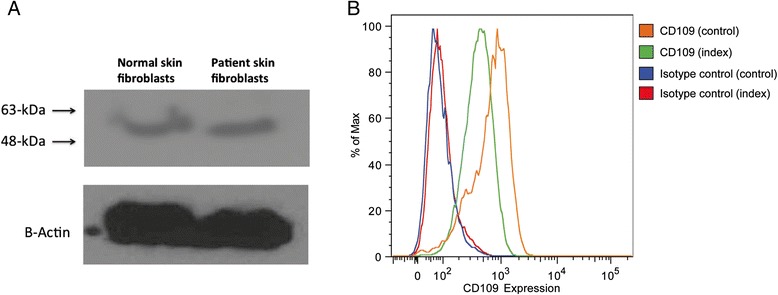
**PIGA expression and surface expression of GPI-AP (CD109) in patient skin fibroblasts. (A)** Western Blot showing similar expression of PIGA in patient versus control fibroblasts; β-actin was used as a loading control. **(B)** Flow cytometry analysis of patient’s fibroblasts revealed reduced expression (56%) of GPI-AP (CD109) when compared to control fibroblasts. Isotype controls are included to show specific binding.

In the WES data we identified four rare homozygous, four rare hemizygous and eight rare compound heterozygous candidates; we did not identify any rare denovo variants affecting protein-coding regions. Of those, only three missense variants were considered functional candidates. *COX7A2* (encoding the nuclear-coded polypeptide chains of cytochrome c oxidase, the terminal oxidase in mitochondrial electron transport (MIM123966)), and *C19orf12* (associated with neurodegeneration with brain iron accumulation 4 (MIM 614298)), and Spastic Paraplegia 43 AR (MIM 615043) phenotypes were subsequently ruled out as Sanger sequencing showed the same recessive genotype in both clinically unaffected brothers. The final candidate, *PIGA* (g.15342986C>T c.989G>A [NM_002641], [p.S330N]) was a novel missense variant not found in more than 250 in-house exomes, dbSNP 138, NHLBI Exome Sequencing Project or Exome Aggregation Consortium (ExAC). The variant is predicted to be the most deleterious of all candidates using the CADD scores [20]. It affects a highly conserved amino acid (Figure 2) and is predicted to be damaging by both PolyPhen-2 [26] and SIFT [22]. The Sanger re-sequencing of the genomic DNA confirmed that index II-2 is hemizygous for the C to T transition, mother is the carrier, while the two unaffected brothers do not have this variant (Figure 1B). Finally, X-inactivation studies showed the pattern of X-inactivation to be 94.5% skewed in the unaffected carrier mother. Together, the genetic analysis based on WES shows that the c.989G>A (p.S330N) variant is a new variant, it affects an evolutionarily conserved amino acid of PIGA resulting in a deleterious change (Figure 2), and segregates with disease in the family (Figure 1B). Functional characterization of the variant showed normal expression of PIGA protein in skin fibroblasts (Figure 3A) but there was a 44% reduction in the surface expression of GPI-anchored CD109 (Figure 3B). The mother of the patient had a normal complete blood cell count and no evidence of red cell hemolysis on peripheral blood smear. A standardized flow cytometric method for screening PNH measuring CD55 and CD59 expression on erythrocytes was negative.

## Discussion

WES revealed a novel, likely pathogenic variant in the *PIGA* gene in our patient with multisystem disease including early onset intractable epilepsy, severe IDD, facial dysmorphism, conductive and sensorineural hearing loss and visual impairment, joint contractures and hepatic and renal involvement.

To date, seven germline mutations in *PIGA* have been discovered using WES technologies in eight unrelated families [7,15-18]. Locations of the pathogenic variants are listed in Table 1 and highlighted on a multi-sequence alignment of PIGA proteins in Figure 2. *PIGA* germline mutations have been found in *XLIDD* male patients with a wide spectrum of clinical diagnoses (Table 1). Mothers of the affected males were confirmed to be carriers in all except for one family (Table 1) where the DNA for the mother was unavailable [18].

**Table 1:** **Overview of mutations and phenotypes observed in patients with germline** ***PIGA*** **mutations**

	**(Johnston et al.** **[** 15 **]** **)**	**(Johnston et al.** **[** 15 **]** **)**	**(van der Crabben et al.** **[** 17 **]** **)**	**(Swoboda et al.** **[** 16 **]** **)**	**(Belet et al.** **[** 7 **]** **)**	**(Kato et al.** **[** 18 **]** **)**	**(Kato et al.** **[** 18 **]** **)**	**(Kato et al.** **[** 18 **]** **)**	**(Kato et al.** **[** 18 **]** **)**	**(Kato et al.** **[** 18 **]** **)**	**Our patient**
	**IV-2**	**IV-4**	**III-1**	**III-9**	**IV-2**	**1**	**2**	**3**	**4**	**5**	
**Mother carrier**	**YES**	**YES**	**YES**	**YES**	**YES**	**Unknown**	**YES**	**YES**	**YES**	**YES**	**YES**
**Mutation**	c.1234C>T [R412*]	c.1234C>T [R412*]	**c.278C>T [P93L]**	**c.328_330delCCT[L344Del]**	c.76dupT [Y26Lfs*3]	c.1234C>T [R412*]	**c.616A>T [I206F]**	**c.230G>T [R77L]**	**c.230G>T [R77L]**	**c.355C>T [R119W]**	**c.989G>A [S330N]**
**Polyhydramnios**	NO	YES	YES	NO	NO	YES	NO	NO	NO	YES	NO
**Current age**	Death at 11wk	Death at 10wk	Death at 2.5 yrs	Death at 7 yrs	24 yrs	6 yrs	10 yrs	8 yrs	18 mo	15 mo	Death at 3.4 yrs
**Sex**	M	M	M	M	M	M	M	M	M	M	M
**Neurology**											
**Developmental delay (severity)**	Early death	Early death	Profound	Profound	Profound	Profound	Profound	Profound	Profound	Profound	Profound
**Hypotonia**	YES	YES	YES	YES	YES	YES	NO	NO	NO	YES	YES
**Hyperreflexia**	YES	YES	NO	YES	NR	NR	NO	NO	NO	YES	YES
**Seizure onset**	Neonatal	Neonatal	8.5 months	7 months	6 months	1 month	3 months	7 months	7 months	3 months	2.4 months
**Seizure types**	Myoclonic	Myoclonic	Generalized clonic (febrile)	Febrile, myoclonic	Myoclonic epileptic seizures	Tonic seizures followed by frequent myoclonus	Myoclonus or epileptic spasm-like movement	Tonic seizures, secondarily generalized seizures	Tonic or clonic	Myoclonic seizures, tonic spasms	Infantile spasms, myoclonic seizures
**EEG findings**	Suppression burst	Suppression burst	Symptomatic generalized epilepsy	Posterior bursts	Hyps-arrhythmia at 7 mo	Suppression burst at neonatal period	Hypsarrhythmia, periodic bursts of multifocal epileptic discharges	Irregular spike and slow wave and multifocal spikes at 2 and5 y	Normal at 7 mo	Hypsarrhythmia at 3 mo, suppression burst at 5 mo	Hypsarrythmia at 2.4 mos, suppression-burst pattern at 3.5 mos
**Seizure prognosis**	Intractable	Intractable	Refractory	Intermittent	NR	Intractable	Intractable	Seizure-free at 3 y with TPM	Seizure-free at 15 mo	Intractable	Intractable
**Thin corpus callosum**	YES	YES	YES	NR	NO	YES	YES	NO	NO	YES	YES
**White matter immaturity**	YES	YES	YES	NR	NO	YES	YES	NO	NO	YES	YES
**Small cerebellum**	YES	YES	YES	YES	NO	NR	NR	NR	NR	NR	YES
**Cortical atrophy**	NR	NR	NR	YES	NO	YES	YES	NO	NO	YES	YES
**Restricted diffusion brainstem/cerebellum**	NR	NR	NR	NR	NO	YES	YES	NO	NO	YES	YES
**Other organs**											
**Facial dysmorphism**	YES	YES	YES	YES	NO	YES	YES	NO	NO	YES	YES
**Joints (contractures)**	YES	YES	NR	NR	NR	YES	YES	NO	NO	NO	YES
**Cardiac**	Systolic II–III/VI murmur with a fixed split S2, ASD	Small PDA	ASD type 2	NR	NR	NR	NR	NR	NR	NR	RVH, arrhythmia (grade 1 AV block, Wenckebach type)
**Liver**	NR	Hepatic microvesicular steatosis	NR	Hepatosplenomegaly	NR	Hepato-megaly, hepato-blastoma	NR	NR	NR	NR	Hepatomegaly, hyperechoic liver
**Kidney**	Vesicoureteral reflux, duplicated collectingSystem	Vesicoureteral reflux	NR	NR	NR	Vesicoureteral reflux	NR	NR	NR	NR	Left hydronephrosis with renal calculi
**Ophthalmologic**	NR	NR	NR	Blindness	NR	NR	NR	NR	NR	NR	Visual motor impairment, retinal dystrophy
**Hearing loss**	NR	NR	NR	Deafness	NR	NR	NR	NR	NR	NR	Sensorineural hearing loss
**Dental**	NR	Underdeveloped gums	Absence of teeth	NR, but III-10 microdontia, widely-spaced, delayed eruption	NR	NR	NR	NR	NR	NR	Microdontia, widely-spaced delayed eruption
**Other**	Globulous chest and small nails, broad palms with short fingers	Absence of olfactory bulb and tracts	Accelerated linear growth, obesity	Ichthyosis	NR	Tracheostomy, micropenis, bilateral inguinal herniation, hypotonic quadriplegia	Spastic quadriplegia, bulbar palsy with gastrostomy and tracheostomy	NR	NR	Transverse palmar crease, prominent calcaneus, left inguinal hernia, hydrocele testicle, hypotonic quadriplegia	Stomatocytes
**Biochemical**											
**Elevated alkaline phosphatase**	NR	YES	YES	NR	NR	NR	YES	NO	NO	YES	YES
**Mitochondrial abnormalities**	NR	NR	Abnormal ATP production (muscle biopsy)	Disorganized mitochondria	NR	NR	NR	NR	NR	NR	Respiratory chain complex I and V reductions (muscle blue native gel)
**Iron storage**	NO	NO	NO	CNS iron deposition	NR	NR	NR	NR	NR	NR	NO
**Other**	Slightly elevated MCV and RDW and low ionized calcium, HgB and RBC	Slightly elevated MCV and RDW	NO	NR	NR	NR	NR	NR	NR	NR	Dyslipidemia (high triglycerides, hypercholesterolemia, LPL deficiency)
**Clinical diagnosis**											
**Clinical descriptor**	MCAHS2 Bethesda– Utrecht syndrome	MCAHS2Bethesda–Utrecht syndrome	MCAHS2 Bethesda– Utrecht syndrome	Ferro-Cerebro-Cutaneous syndrome	MCAHS2-like syndrome X-linked infantile spasm syndrome (West syndrome)	Ohtahara syndrome, early myoclonic encephalopathy, Schinzel-Giedion syndrome	West syndrome with hypomyelination	Early-onset epileptic encephalopathy	Early-onset epileptic encephalopathy	West syndrome	PIGA deficiency
**Cause of death**	Pneumonia	Respiratory failure	Cardiac arrest	Aspiration pneumonia	NA	NA	NA	NA	NA	NA	Cardiac arrest

The phenotypic spectrum of *PIGA* germline mutations has shown wide variation, as reflected by the range of clinical diagnoses summarized in Table 1. However, a common set of characteristic features, shared by our patient, has emerged including infantile spasms with hypsarrhythmia on EEG (Table 1) and IDD. Generally, the phenotypic spectrum could be classified into two types (severe and less severe), as proposed by Kato et al. [18], where presence of facial dysmorphism correlates very well with the more severe spectrum of clinical manifestations (Table 1). In addition to early onset infantile spasms with hypsarrhythmia on EEG, IDD and profound developmental delay, the patients with more severe manifestations of *PIGA* germline mutations (patients: IV-2, IV-4 [15], III-1 [17], III-9 [16], 1, 2, and 5 [18], and our case [this report]) also present with dysmorphic facial features, multiple CNS abnormalities, such as thin corpus callosum and delayed myelination, as well as hypotonia (Table 1). Other phenotypes such as polyhydramnios, joint contractures, hyperreflexia, cardiac anomaly, severe developmental delay and elevated alkaline phosphatase are also recurrently seen in these patients, while additional phenotypes appear to be allele-specific (Table 1). Unlike patients with severe phenotypes, the less severe form of *PIGA* germline mutations (patients: IV-2 [7], 3 and 4 [18]) did not involve facial dysmorphism and multiple CNS abnormalities, but did present with early onset of infantile spasms with hypsarrhythmia on EEG (Table 1), and generally longer life span marked by IDD, treatable seizures and PDD (Table 1).

Early onset infantile spasms appear to be a common feature of *PIGA* mutations, as is seen with many other defects in GPI-anchor biosynthesis. Chiyonobu et al. [27] hypothesized that these seizures are due to intracellular pyridoxal phosphate deficiency secondary to the loss of membrane bound alkaline phosphatase, which is required to initiate pyridoxal-phosphate for transit of the plasma membrane (after which it is rephosphorylated). Given that GABA synthase requires intracellular pyridoxal-phosphate, it is the intracellular GABA deficiency which likely leads to the onset of seizures [27].

Additional features are frequently reported in patients with the severe form of *PIGA* germline mutations, as proposed by [18] including: hypotonia (7/8 patients with available data), elevated alkaline phosphatase (5/5 patients with available data), hyperreflexia (5/5 patients with available data), joint contractures (5/6 patients with available data), cardiac anomalies (4/4 patients with available data) and polyhydramnios (4/8 patients with available data) possibly due to dysphagia associated with hypotonia. The plagiocephaly facial dysmorphism in our and published cases is likely secondary to the hypotonia, causing facial asymmetry and distortion of proportions as well as secondary contractures. Our patient specifically appears to have a short nose with anteverted nares and a depressed nasal bridge, seen as the triangular structure at the base of the nose (Figure 1A). This feature appears to be present in the more severely affected published cases [18]. Additional features such as dental problems (e.g. microdontia, delayed eruption) [16,17], liver problems [15,16,18] visual impairment and deafness [16] appear to be recurrent as well (Table 1). On the other hand some features, such as accelerated linear growth and obesity [17], CNS iron deposition and ichthyosis [16], dyslipidemia and stomatocytosis (in the patient reported here) are reported only once. The cause of death in patients with *PIGA* mutations has been mainly due to cardiac arrest, pneumonia and respiratory failure (Table 1). Although the life span of the patients affected with less severe mutations is generally longer, there is a great degree of variability in life-span even in patients with the same germline mutation [15,16,18,28].

Reports of brain MRI findings in PIGA deficiency describe white matter immaturity with insufficient myelination, cerebral atrophy, thinning of the corpus callosum, and a small cerebellum [7,15,18]. Cerebral atrophy appears to progress rapidly and is associated with abnormal white matter myelination, consistent with an early neurodegenerative process [18]. Our case showed a similarly rapid progression, with an established cerebral atrophy associated with disrupted subcortical myelination at age 2.5 years. However, the most remarkable finding in the early stages of the disease was restricted diffusion in the brainstem tegmentum, superior cerebellar peduncles, subthalamus, and ventral striatum, indicative of intramyelin edema, involving selectively white matter regions that are already physiologically myelinated at birth. Kato et al. [18] also reported three cases showing restricted diffusion at the brainstem, basal ganglia, thalamus, and deep white matter. Interestingly, these findings are remarkably similar to those of the classical form of MSUD, an amino aciduria caused by deficiency of branched chain α-keto acid dehydrogenase enzyme, in which brain MRI of affected newborns or infants studied during stages of metabolic decompensation shows prominent signal changes and swelling within myelinated brain areas representing intramyelin edema, caused by a deficit of Na+/K+ ATPase activity as a result of impairment in energy production secondary to branched chain amino acids accumulation [29,30]. In particular, the findings of our case are remarkably similar to those shown in figure six in the report by Rossi and Biancheri [31], implicating that the diagnosis of PIGA deficiency could be suggested in neonates with similar brain MRI findings and unremarkable plasma amino acid and urine organic acid profiles. An explanation for the similarities remains speculative; the typical lesions in the newborn with MSUD involve preferentially those structures that are already myelinated at birth and thus with higher metabolic demands and most vulnerable to the energy deficiency and toxicity of branched-chain 2-oxo acid(s). In PIGA deficiency, although toxins have not yet been identified, we speculate that mitochondrial and thus energy deficiency could similarly play a role in the etiology of the observed intra-myelin edema. Regardless of the implicated pathophysiologic mechanism, identification of similarities in the lesion distribution and appearance on MRI represents the foundation of the pattern recognition approach, whereby neuro-imaging can guide the diagnostic process to reduce the number of unnecessary tests and time to diagnosis [32]. PIGA catalyzes the first step in GPI biosynthesis and it is anticipated that even a partial defect in activity will have a significant impact on the localization and functionality of a broad range of GPI-anchored proteins. The elevation in serum alkaline phosphatase reported here as well as in the majority of other PIGA deficient cases (likely correlated with severity of the disease) (Table 1) as well as other PIG deficiencies results from the secretion of GPI-deficient alkaline phosphatase (normally membrane-anchored) and may serve as a diagnostic clue. Normal levels of alkaline phosphatase however, do not rule out the condition, as evidenced by the mild case reported by Belet et al. [7]. The hypertriglyceridemia and serum LPL deficiency observed in our patient is also likely caused by abnormal GPI biosynthesis. LPL release from endothelial cells in response to heparin stimulation requires the activity of GPIHBP1 (GPI-anchored high-density lipoprotein binding protein 1) and primary genetic defects in this GPI-anchored protein lead to hypertriglyceridemia and non-detectable serum lipoprotein lipase [33]. The GPI biosynthesis defect resulting from *PIGA* mutation likely leads to a secondary GPIHBP1 deficiency, although further evaluation is required to confirm this mechanism, as a similar defect has not been described or investigated in other individuals with PIGA defects.

The germline mutational spectrum for *PIGA* comprises various mutation types including one nonsense mutation [15,18], one frameshift mutation that results in production of 36 amino acids shorter PIGA protein [7], one small in-frame deletion [16], and four missense mutations [17,18] (Figure 2). PIGA protein is well conserved from yeast to human (Figure 2), and all of the reported mutations, except for c.76dupT, occurred at evolutionarily conserved or semi-conserved amino acids (Figure 2). The c.76dupT frameshift mutation occurs in the non-conserved part of the protein but results in translation of the PIGA protein using a cryptic start site at amino acid position 37 producing shorter PIGA protein with the majority of the conserved amino acids intact [7]. One mutation, c.1234C>T, was recurrent (Table 1) [15,18].

Studies in mice revealed that complete disruption of the *PIGA* gene results in early embryonic lethality in males, while in carrier female mice late embryonic lethality is observed [3]. Based on these studies, it is believed that complete loss of PIGA function is lethal in humans. A number of studies suggest that the human mutations identified to date result in reduced, but not absent, PIGA activity and using the flow cytometry of blood granulocytes method, Kato et al. [18] showed that the phenotype severity of the *PIGA* germline mutations appeared to correlate with genotype and the residual functional activity of the PIGA protein [18]. Functional studies on the truncating c.1234C>T mutation (p.R412X) suggest that small amounts of full length PIGA protein were generated by the read through of the premature termination codon (PTC) [15,18]. During two stages of eukaryotic translation (elongation and termination), aminoacyl-tRNAs and termination factors compete for codon binding. When aminoacyl-tRNAs supersedes, read through of the termination codon occurs, which allows the generation of the full-length polypeptide. Depending on the amino acid inserted during the read through, the resulting protein may have normal or partial activity. Studies on base level of read through reveal 10 fold higher frequency at PTCs (<1%) [34,35] when compared to naturally occurring stop codons (<0.1%) [36,37]. In addition to c.1234C>T (p.R412X), studies on the c.328_330delCCT (p.L344Del) mutation revealed reduced GPI-anchored proteins on the patient’s granulocytes, while normal levels were observed on the red blood cells and monocytes, suggesting reduced but not absent PIGA activity [16]. Complementation assays using the c.76dupT frameshift mutation confirmed partial function of the shorter PIGA protein, which was sufficient to rescue surface expression of CD59 in a PIGA null cell line [7]. Consistent with previous findings, our patient’s mutation also led to reduced surface expression of the GPI-anchored protein CD109 on skin fibroblasts, despite normal levels of protein expression.

Patients with *PIGA* germline mutations share key phenotypic features with patients carrying mutations in genes encoding various PIG family members, including IDD, seizures, hypotonia, growth defects, congenital abnormalities, heart defects, and abnormal metabolic profiles. For example, inherited glycosylphophatidylinositol deficiency (MIM 610293) resulting in portal- and hepatic-vein thrombosis and absence seizures was found to be due to mutations in *PIGM* gene (MIM610273) [5]. Hyperphosphatasia Mental Retardation syndrome (HPMRS, MIM 239300, MIM 214749, and MIM 614207), also known as Mabry syndrome, was found to be associated with *PIGV* (MIM 610274), *PIGO* (MIM 614730),*PGAP2* (MIM 615187) and *PGAP3* (MIM 611801) mutations [38-42]. HPMRS is characterized by elevated alkaline phosphatase levels, IDD, seizures, hypotonia, and facial dysmorphic features [38]. CHIME syndrome (MIM 280000), also known as Zunich Neuro-Ectodermal syndrome was found to be due to mutations in *PIGL* (MIM 605947). Individuals with CHIME syndrome present with colobomas, congenital heart defects, early onset migratory ichthyosiform dermatosis, IDD, and ear anomalies, including conductive and sensori-neural hearing loss [43]. *PIGT* mutations cause congenital anomalies-seizures-hypotonia type 3, with hypophosphatasia as key feature [44,45]. The wide spectrum of human conditions associated with mutations in PIG genes reflects their role in multiple developmental processes and resembles the diversity of clinical features associated with glycosylation pathways deficiencies [43].

A variety of clinical case descriptors have been applied to individuals with germline *PIGA* mutations (Table 1). However, given the varied clinical spectrum reported to date, it appears that the multi-system roles of GPI-anchored proteins and private nature of most of the PIGA mutations identified will preclude the definition of a common feature set. To unify further reports on this intriguing condition, we advocate for the use of ‘PIGA deficiency’.

Careful patient phenotyping will continue to shed light on the (patho-) physiologic roles of PIGA deficiency; for example, its relation with mitochondrial structure and function. We are the first to report severely reduced amount of mitochondrial complexes (specifically I and V), but other mitochondrial defects have been reported in *PIGA* mutations: e.g. ‘disorganized mitochondria’ in FCC syndrome [16] and ‘abnormal ATP production’ in a patient with accelerated linear growth and obesity [17]. In fact, recent publications show that several mitochondrial membrane proteins are either modified with GPI anchor addition or associated with GPI anchored proteins, a process required for their proper function [46]. Therefore, it is of great interest to test patients with the *PIGA* mutations for mitochondrial defects and *vice versa*, to test the patients with unexplained mitochondrial phenotypes for potential *PIGA* mutations, especially in the presence of features described here.

## Conclusions

Based on the patient descriptions published to date, intractable, infantile onset epilepsy with suppression burst and/or hypsarrhythmia in patients with X-linked IDD of unknown etiology should prompt clinicians to consider germline mutations in the *PIGA*, particularly in a patient with elevated serum alkaline phosphatase. The additional presence of dysmorphic facial features, CNS abnormalities, hypotonia, heart defects, dyslipidemia/lipoprotein lipase deficiency, or signs of intra-myelin edema on brain MRI, may increase the likelihood of mutations in *PIGA* specifically, and *PIG* family members in general. Flow cytometry of blood granulocytes has also proven a useful test for levels of GPI-anchored proteins expression in patients with *PIGA* mutations [7,15,16,18].

## Abbreviations

PIGA: Phosphatidylinositol glycan biosynthesis class A protein
GPI: Glycosylphosphatidylinositol
PIG: Phosphatidylinositol glycan biosynthesis protein
PI: Phosphatidylinositol
PNH: Paroxysmal nocturnal hemoglobinuria
GlcNAc: N-acetylglucosamine
UDP-GlcNAc: UDP-N-acetylglucosamide
WES: Whole Exome Sequencing
MSUD: Maple syrup urine disease
MCAHS2: Multiple congenital anomalies-hypotonia-seizures syndrome 2
FCCS: Ferro-Cerebro-Cutaneous syndrome
BNG: Blue Native Gel
OFC: Occipital frontal circumference
NAA: *N*-Acetylaspartic acid
TORCH: Toxoplasmosis, rubella, cytomegalovirus, herpes simplex, and HIV
IDD: Intellectual developmental disorder
TIDEX: Treatable Intellectual Disability Endeavour eXome sequencing
CNV: Copy number variation
CADD: Combined Annotation–Dependent Depletion
SIFT: Sorting Intolerant From Tolerant
AR: Androgen Receptor
XCI: X-Chromosome inactivation
XLIDD: X-linked intellectual developmental disability
GPIHBP1: GPI-anchored high-density lipoprotein binding protein 1
HPMRS: Hyperphosphatasia Mental Retardation syndrome
PTC: Premature termination codon
